# Long COVID headache

**DOI:** 10.1186/s10194-022-01450-8

**Published:** 2022-08-01

**Authors:** Claudio Tana, Enrico Bentivegna, Soo-Jin Cho, Andrea M. Harriott, David García-Azorín, Alejandro Labastida-Ramirez, Raffaele Ornello, Bianca Raffaelli, Eloísa Rubio Beltrán, Ruth Ruscheweyh, Paolo Martelletti

**Affiliations:** 1 Center of Excellence On Headache, Geriatrics and COVID-19 Clinic, SS Annunziata Hospital of Chieti, 66100 Chieti, Italy; 2 Internal Medicine and Emergency Medicine, Sant’ Andrea Hospital, Sapienza University, Rome, Italy; 3 Department of Neurology, Dongtan Sacred Heart Hospital, Hallym University College of Medicine, Hwaseong, Republic of Korea; 4 Headache and Neuropathic Pain Unit, Massachusetts General Hospital, Harvard Medical School, Boston, MA USA; 5 Headache Unit, Department of Neurology, Hospital Clínico Universitario de Valladolid, Valladolid, Spain; 6 Headache Group, Wolfson Center for Age Related Diseases, Institute of Psychiatry, Psychology and Neuroscience, King’s College London, London, UK; 7 Departement of Applied Clinical Sciences and Biotechnology, University of L’Aquila, L’Aquila, Italy; 8 Department of Neurology, Charité Universitätsmedizin Berlin, Berlin, Germany; 9 Department of Neurology, Ludwig Maximilians University, Munich, Germany; 10 German Migraine and Headache Society, Frankfurt, Germany; 11 Department of Psychosomatic Medicine and Psychotherapy, Technical University of Munich, Munich, Germany

**Keywords:** Headache, Coronavirus disease-19, SARS-CoV-2, Migraine, Diagnosis, Pain, Morbidity

## Abstract

Headache is among the most frequent symptoms persisting or newly developing after coronavirus disease 2019 (COVID-19) as part of the so-called long COVID syndrome. The knowledge on long COVID headache is still limited, however growing evidence is defining the features of this novel condition, in particular regarding clinical characteristics, some pathophysiological mechanisms and first treatment recommendations. Long COVID headache can present in the form of worsening of a preexisting primary headache, or, more specifically, in the form of a new (intermittent or daily) headache starting during the acute infection or after a delay. It often presents together with other long COVID symptoms, most frequently with hyposmia. It can manifest with a migrainous or, more frequently, with a tension-type-like phenotype. Persistent activation of the immune system and trigeminovascular activation are thought to play a role. As there are virtually no treatment studies, treatment currently is largely guided by the existing guidelines for primary headaches with the corresponding phenotype. The present report, a collaborative work of the international group of the Junior Editorial Board of The Journal of Headache and Pain aims to summarize the most recent evidence about long COVID headache and suggests approaches to the diagnosis and treatment of this disorder.

**Graphical Abstract:**

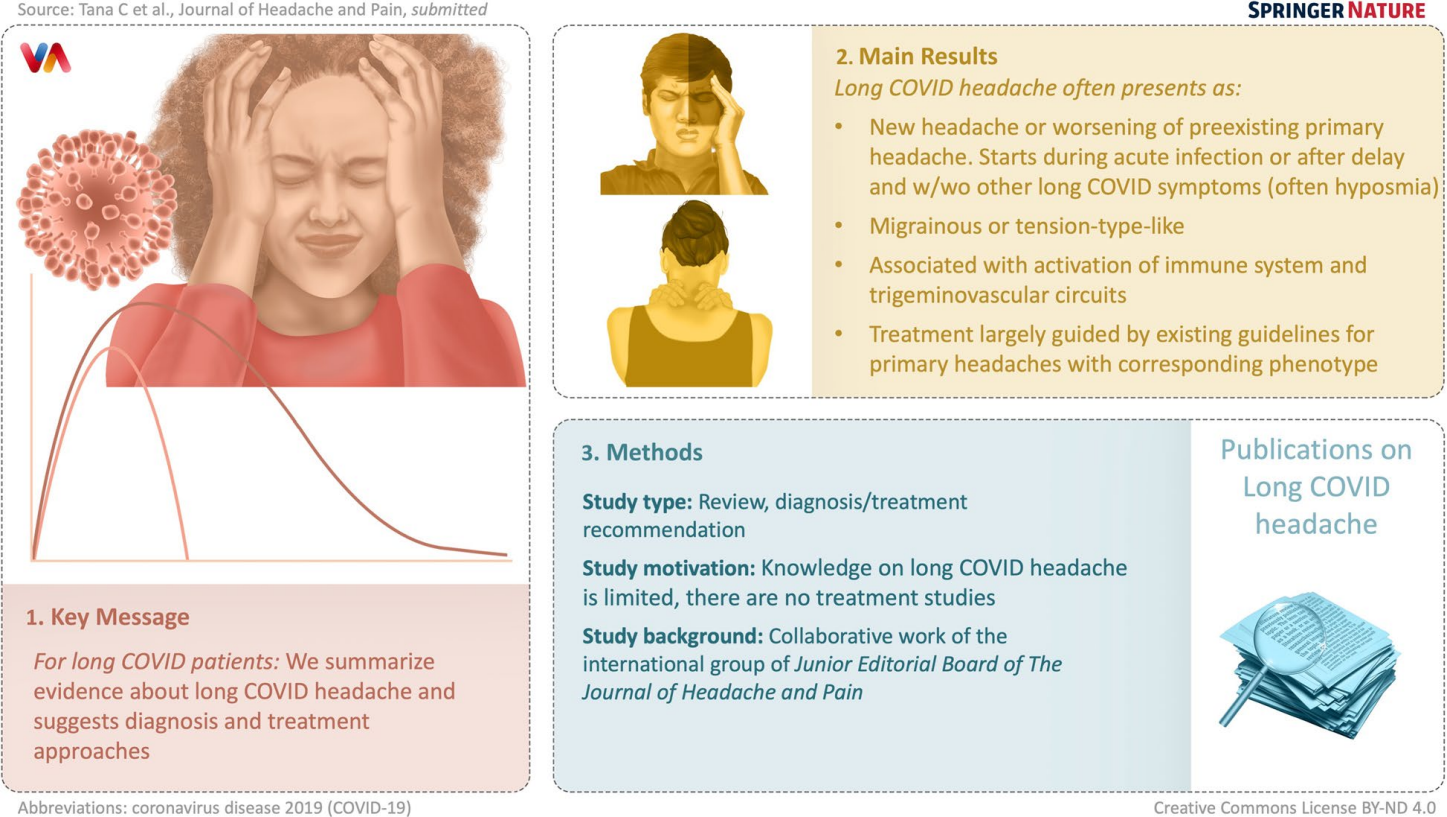

## Introduction

The years 2020–2022 have been marked by a severe pandemic from a novel coronavirus disease (COVID-19) which affects mainly the respiratory system. Before the development of an effective vaccination campaign which led to the immunization of a large part of the world population, COVID-19 was associated with a high risk of mortality, mainly due to acute respiratory failure [1–3]. Moreover, part of the people who recovered from acute COVID-19 suffer from a spectrum of symptoms persisting for weeks and even months after the acute infection is gone. This syndrome is characterized by a wide range of health problems including “brain fog” with cognitive disturbances, fatigue, dyspnea, myalgia and muscle weakness, depression, and persistent headache [4]. Different names have been used to describe the syndrome, among them post-COVID-19 syndrome, post-COVID condition, COVID-19 consequences and post-acute sequelae of SARS CoV-2 infection. Here, we adopt the term long COVID in recognition of the patient-researchers who first described the entity in spring of 2020 [5].

Headache can be one of the most disabling symptoms of long COVID and may manifest alone or in combination with other symptoms. The burden, characteristics, pathophysiology and management of long COVID headache are not completely understood. However, growing evidence is defining the features of this novel condition, in particular regarding clinical characteristics, some pathophysiological mechanisms and first treatment recommendations. The present report, a collaborative work of the international group of Junior Editorial Board of The Journal of Headache and Pain aims to summarize the most recent evidence about long COVID headache and suggests approaches to the diagnosis and treatment to this disorder. Future directions of study will be discussed at the end, focusing on those aspects that could be useful in the daily clinical approach to patients with long COVID headache.

## Definition, classification and epidemiology

National and international health organizations have proposed several definitions to describe prolonged symptoms following COVID-19 (Table 1) [6–8]. The United States have more than 80 million patients and survivors of COVID-19, which is the highest number in the world, and main healthcare organizations have aimed at giving a definition to the emerging issue. However, there is currently no consensus about the definition of the long COVID syndrome. Especially, the time frame used to define the long COVID is not clear. Usually, at least 4 weeks after diagnosis or onset of acute COVID-19 symptoms are required before calling symptoms persistent. One article has proposed a classification into three different phases, weeks 5–12, week 12–24 and > week 24 after diagnosis [9]. The National Institute for Health and Care Excellence (NICE) has distinguished between ongoing symptomatic COVID-19 and post-COVID-19 syndrome for people who have symptoms respectively between 4 and 12 weeks and more than 12 weeks after the onset of acute disease [10]. Studies have been conducted mainly in adult subjects, therefore the evidence of long COVID in children is very limited [9, 10]. The International Classification of Headache disorders uses a headache duration of > 3 months after the acute infection for the diagnosis of “Chronic headache attributed to systemic viral infection” [11] but it must be considered that using a 3 months time frame may lead to delay of necessary treatment.

**Table 1:** Reports some definitions of long COVID by the most important healthcare organizations

International society, year	Definition of long COVID
Centers for Disease Control and Prevention (CDC, 2021)	“Wide range of new, returning, or ongoing health problems people can experience four or more weeks after first being infected with the virus that causes COVID-19”
World Health Organization (WHO, 2021)	“Illness that occurs in people who have a history of probable or confirmed SARS-CoV-2 infection, usually within three months from the onset of COVID-19, with symptoms and effect that last for at least two months, that cannot be explained by an alternative diagnosis”
National Health Service (NHS, 2021)	“Symptoms lasting weeks or months after the infection has gone”
National Institute for Health and Care Excellence (NICE, 2021)	“Two definitions of postacute COVID-19 are given: (1) ongoing symptomatic COVID-19 for people who still have symptoms between 4 and 12 weeks after the start of acute symptoms; and (2) post-COVID-19 syndrome for people who still have symptoms for more than 12 weeks after the start of acute symptoms.”

There are currently a total of over 500 million confirmed cases of COVID-19 worldwide [12]. Headache is one of the earliest and most common symptoms during the acute phase of COVID-19; characteristically it appears as oppressive pain in the upper/frontal part of the head and affects between 14 and 60% of patients during the acute COVID-19 phase [13, 14]. Headache, with a prevalence of 18%, seems to be the fifth most common symptom in patients with long COVID after fatigue, dyspnea, myalgia and cough. Another possible long-term manifestation of pneumonia from SARS-CoV-2 infection is lung fibrosis [15]. Some studies have underlined how neurological and psychological symptoms seem to cluster and be more common in some types of patients: a cross-sectional study found persistent headache in 50% of patients who experienced hyposmia after months of recovery from SARS-CoV-2 infection suggesting a common pathophysiological substrate [16–18].

The problems of long-lasting symptoms after viral infections are not unique to COVID-19, and the recent reduction of mortality resulting from the vaccination from SARS-CoV-2 and from apparition of the less pathogenic omicron variant has been associated with a significant increase of survival rate and of patients who experience persistent symptoms after SARS-CoV-2 infection [19]. Therefore, long COVID is progressively serious issue worldwide.

Long COVID headache can be classified according to the clinical presentation or phenotype, associated symptoms, virus variant, or to the diagnostic criteria of the International Classification of Headache Disorders, 3rd edition (ICHD-3) [11]. Regarding clinical presentation, long COVID headache can manifest with a clinical picture similar to that of the new daily persistent headache, classified as NDPH in the ICHD-3, because of its prominent temporal relationship and resistance to treatment [11]. However not all long COVID headaches have daily frequency, and may be affected by the disease severity and use of analgesics, so it may present as intermittent or chronic daily headaches. Chronic daily headache is also associated with a significant burden in terms of functional impairment and psychological comorbidities. Actual prevalence of a NDPH-like or chronic daily headache after the onset of COVID-19 has not been yet reported [20]. According to the ICHD-3 classification, most headaches from COVID-19 can be classified as headaches attributed to systemic viral infection and, like other secondary headaches, they are characterized by bilateral and pressing quality, and the phenotype of tension-type headache is more common than the migrainous one, as mentioned before [21].

Most patients complain of various symptoms other than headaches, and symptoms (and therefore the long COVID phenotype) can vary according to the type of virus variant (e.g. Alpha, Delta, Omicron). Fever, cough, and loss of taste were reported as common symptoms for the Alpha variant, while runny nose, headache, and fatigue were reported more often for the Omicron type. [22–24].

## Risk factors and pathogenesis

The frequency of long COVID headache seems to be similar in patients with severe and non-severe forms of COVID-19 disease. In a meta-analysis which evaluated 35 studies up to May 2021, accounting for 28,348 COVID-19 survivors, the prevalence of post-COVID headache was higher in patients that were managed in an outpatient setting during the acute phase, but not at 30, 60 or 90 days [18]. A prospective study that assessed data from six cohorts for nine months, including 905 headache patients out of 3698 COVID-19 cases during the acute phase of disease, found the frequency of headache over time was similar in patients managed in an outpatient setting as compared to those who were hospitalized. However, in patients who had persistent headache at 9 months (15.4%), the frequency of pneumonia during the acute phase was lower (36.4% vs. 47.9%). In this study, patients with persistent headache at 9 months were older (52 vs. 47 years) and more frequently female (75.7 vs. 66.1%) [25]. Low information are available about differences between headache characteristics in patients following ICU admission for severe Sars-CoV-2 infection or in those cases treated at home, or in patients having longer hospitalization, who were ventilated or not.

### Trigeminovascular system activation in patients with genetic predisposition to migraine or pre-existing headache

It has been hypothesized that long COVID headache could emerge as a result of the “activation” of a pre-existing headache or in patients with a genetic predisposition to migraine by activating the trigeminovascular system. In the previously mentioned study, some migraine-like features of the headache during the acute phase were associated with long COVID headache. The phenotypic headache variables during an infection from SARS-CoV-2 were associated with a higher frequency of persistence of headache at 9 months, included higher frequency of throbbing quality (reported in 40.6% during the acute phase in patients with persistent headache at 9 months vs. 17.3%), lower frequency of pressing quality (40.6% vs. 63.4%), higher frequency of photophobia / phonophobia (45.7% vs. 34%) and higher frequency of worsening by physical activity (45.7% vs. 34%) [18]. In a multicentric study including 615 patients of which 205 having headache during the acute phase of COVID-19, followed for a mean of 7.3 months, the presence of headache during COVID-19 was associated with a higher frequency of long COVID symptoms (2.4 vs. 2.0 symptoms), higher frequency of fatigue during long COVID (OR: 1.55; 95% CI: 1.07–2.24) and a higher frequency of tension-type-like headache phenotype (2.65; 95% CI: 1.66–4.24). In that sample, prior history of migraine was also associated with post-COVID headache (OR: 2.90; 95% CI: 1.41–5.98) [25, 26]. These data have been previously explored in a smaller sample from the same cohort, that evaluated 57 patients with confirmed diagnosis of migraine prior to the COVID infection and 144 age-and-sex matched controls, where no significant differences were observed regarding anxiety or depression during long COVID evaluated with the Hospital Anxiety and Depression Scale (HADS), but with a higher frequency of post-COVID symptoms (OR: 1.50; 95% CI: 1.09–2.09) [27]. Another case–control study observed that patients with prior history of migraine had 40% more frequent long-lasting headache, as defined by continuous headache present for more than 1 day [28].

### Immune system activation

Acute headache attributed to a systemic viral infection may be related with a significant systemic immune response [11]. The presence of headache during the acute phase has been associated with a better prognosis, including lower mortality, lower duration of the acute phase and a lower need for intensive care unit admission [29–31]. This has been associated with a more efficient immune response. Indeed, patients with headache had lower blood levels of D-dimer, C-reactive protein, lactate dehydrogenase, ferritine, and higher levels of lympocythes [29–31].

It has been hypothesized instead that patients with long COVID headache can manifest a persistent immune system activation with biohumoral response, as demonstrated by the evidence of altered blood levels of cytokines and interleukins. Two studies observed lower levels of interleukin-6 [30, 31], while another study observed higher mean levels of IL-6, albeit differences were not statistically significant. In that study, the subgroup of patients with bilateral headache (77 out of 83 patients with headache), had higher levels of IL-6 than patients with unilateral headache [32]. The same group published another case–control study including 88 patients, where serum levels of HMGB1, NLRP 3, IL-6, angiotensin II and ACE 2 were higher in patients with headache during COVID-19 [33]. Another group found a significant alteration of IL-10 blood levels, after an analysis of 45 different cytokines and interleukins in 104 patients at the time of their emergency department visit [34]. Although the hypothesis of a persistent activation of the immune system in patients with predisposing headache biology could be supported by some data, the direct evidence about the immune response over time in terms of years in patients with long COVID headache is limited. A multivariate analysis of 576 hospitalized patients that were subsequently followed for one year, observed that immune-compromised patients had a more prolonged duration of the headache over time (HR: 2.9; 95% CI: 1.02–8.22) [35].

### Hypoxia and/or hypercapnia

The first theories that were considered regarding COVID-19 headache pathophysiology were hypoxia and/or hypercapnia [36]. This hypothesis was evaluated in a cohort of 70 patients and followed 3 months after the acute phase. There were no differences regarding cardio-pulmonary function, assessed by laboratory parameters, echocardiography, pulmonary function tests or cardio-pulmonary exercise test [37].

### Structural and functional brain changes

**Fig. 1:**
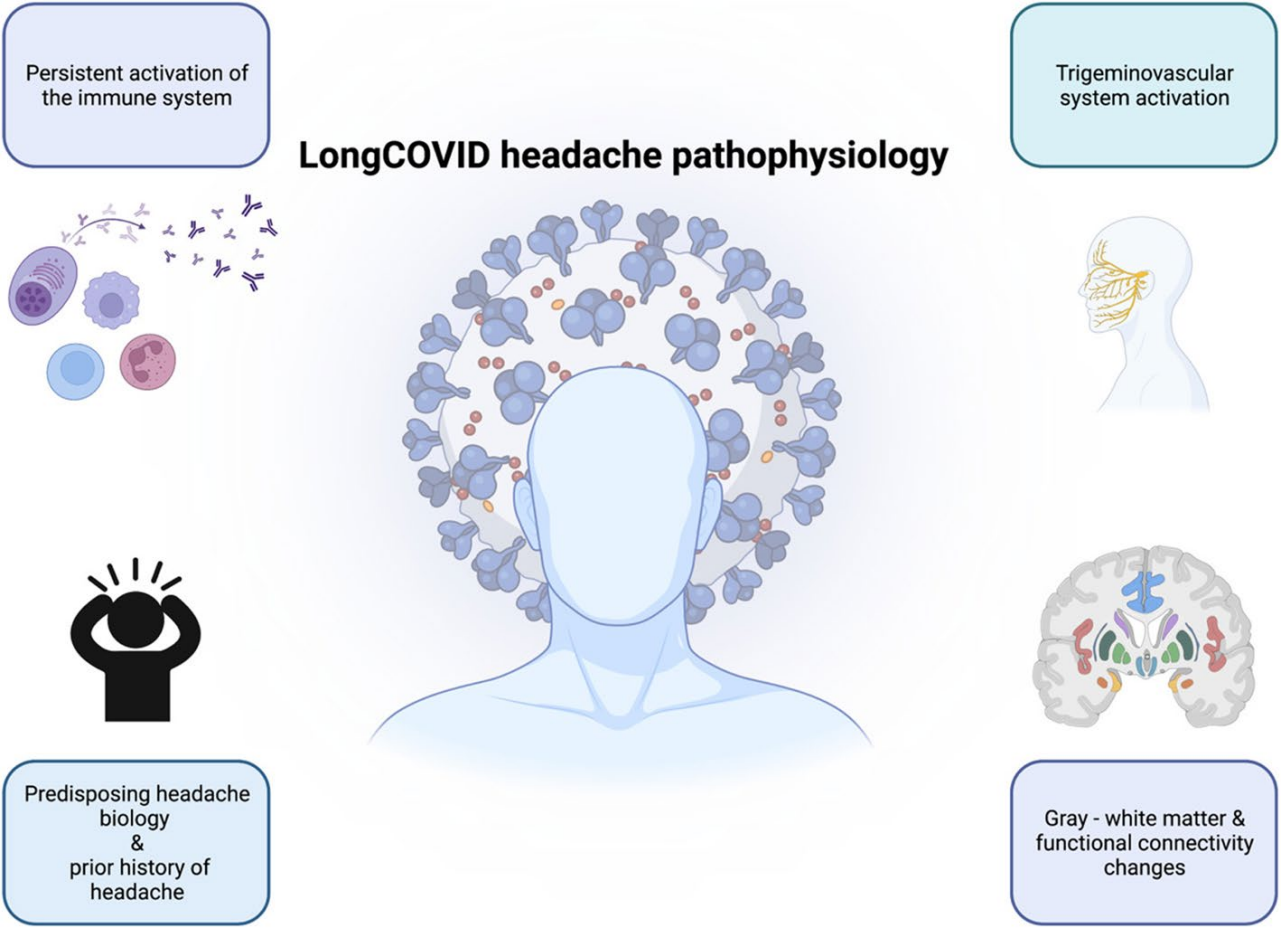
Main pathophysiological mechanisms which have been hypothesized for long COVID headache

Some neurological manifestations of long COVID may be associated with structural and functional brain changes. A longitudinal project that was studying brain structure and cognitive function over time in 785 participants prior to the pandemic onset was used to assess COVID-19 effects. The availability of neuropsychological and MRI information allowed the comparison between patients that tested positive for COVID-19 (*n* = 401) and participants that remained COVID-19 free (*n* = 384). A second comprehensive evaluation, including MRI, was done 38 months in mean after the first one. In that assessment, gray matter reduction was observed in patients with COVID-19, specifically in orbitofrontal cortex and parahippocampal gyrus [38]. Furthermore, a higher cortical surface area and gray matter volume in orbitofrontal cortex was observed in a sample of patients with long COVID headache, but no differences were documented regarding cortical thickness [39], which could suggest that some gray matter changes may be manifestation-specific. Indeed, most changes were observed in areas that were functionally connected with the primary olfactory cortex [36]. Resting-state functional connectivity has been compared between COVID-19 survivors and healthy controls, observing weakened functional connections between the cingulate, hippocampal gyri, parietal, temporal and frontal gyri and strengthened functional connectivity with occipital regions [39]. Besides gray matter and connectivity changes, white matter changes have been reported in COVID-19 survivors, compared with healthy controls, with higher axial diffusivity in corona radiata, and internal and external capsules [39, 40], suggesting some degree of white matter axonal alterations, which could be involved in the persistence of headache, however, the specificity of these changes in regards to headache is still to be ascertained. Some of these changes could be related with COVID-19 and its comorbidities, or specifically with headache, so whether the observed brain changes are cause or consequence of headache is currently unknown. Concerning brain metabolism, there are no headache-specific studies, but the regions most frequently reported as hypometabolic include the right parahyppocampal gyrus [41], the brainstem, thalamus, amygdala [41], orbital gyrus, olfactory gyrus, and temporal lobe [42]. One study compared images from 18-fluor-deoxy-glucose PET and functional MRI imaging, showing an overlap between the areas where the connectivity was altered in the MRI and F-18 FDG PET changes [43]. Figure 1 summarizes the main mechanisms which could be involved in the long COVID headache pathophysiology.

## Clinical features and diagnosis

Headache is one of the most frequent neurologic symptoms associated with long COVID syndrome [44, 45]. Long COVID headache is more prevalent in middle-aged women and it is characterized by a moderate to severe intensity headache which can be accompanied by concomitant long COVID symptoms such as fatigue, cognitive dysfunction and dizziness, as well as hyposmia and insomnia or other sleep impairments [25, 30].

Long COVID headache does not seem to have a specific clinical phenotype. A limited number of cross-sectional studies have revealed that the topography of the headache is predominantly bilateral with frontal or periocular predominance and oppressive quality [25, 30]. However, the headache phenotype is highly variable as temporal and occipital predominance and throbbing quality is also frequently seen in approximately one third of patients [25, 30].

Although the described clinical presentation is heterogeneous and might involve a wide spectrum of headache presentations [46], long COVID headache usually mimics primary headaches phenotypes. The two most frequent phenotypes described are tension-type-like and migraine-like headache, whereas cluster headache-like phenotype has been rarely documented [25, 30]. Migraine-like headaches can be accompanied by nausea, vomiting, photophobia, and phonophobia, and can be aggravated by routine physical activity, whereas tension-type headaches are not aggravated by routine physical activity and are rarely associated with other symptoms [25, 46].

Patients with a prior history of headache usually report a worsening of their headache [25, 30]. Interestingly, both tension-type-like and migraine-like headache features can also be seen in patients without a personal headache history or despite not having experienced headache in the acute infection phase [25, 30]. Another factor associated with long COVID headache is treatment resistant headaches during the acute phase of infection [47].

Due to the novelty and limited information available of long COVID headaches, further large population multicentre studies are required to: (1) fully characterize the headache phenotype specifically of long COVID headache (not acute COVID headache); (2) establish long-term disability and impact on the patient's quality of life; and (3) determine the risk of chronicization of pre-existing headaches.

### Diagnosis of long COVID headache

As mentioned above long COVID headache does not have a specific clinical presentation, therefore the diagnosis of such a headache disorder is mainly a diagnosis of exclusion. ‘Pure’ long COVID headache should be differentiated from the exacerbations of preexisting headache disorders, and mostly migraine. Headache associated to long COVID usually has tension-type features; however, one quarter of cases have migraine-like features [30, 48]. However, pre-existing low-frequency episodic migraine or pure menstrual migraine can be overlooked in the general population [49]. A preexisting low-frequency migraine could be exacerbated by COVID-related systemic inflammation [50, 51] and mimic a new-onset headache.

In the absence of a previous history of headache disorders, the new onset of headaches should be differentiated from other primary and secondary headache disorders. Interestingly, COVID-19 infection might generate headache episodes clinically indistinguishable from migraines even in subjects who do not suffer from migraine [30]. Patient history is key to determine whether the onset of a new headache is attributable to long COVID infection. Age and sex should be considered, as COVID-19 infection could precede the onset of migraine in young women. Headache symptoms are very prevalent in the general population and might not be necessarily related to COVID-19 infection. Another important clue is the presence of other COVID-related symptoms such as ageusia and anosmia, which indicate nervous system invasion and are strongly correlated with Covid-related headache.

**Fig. 2:**
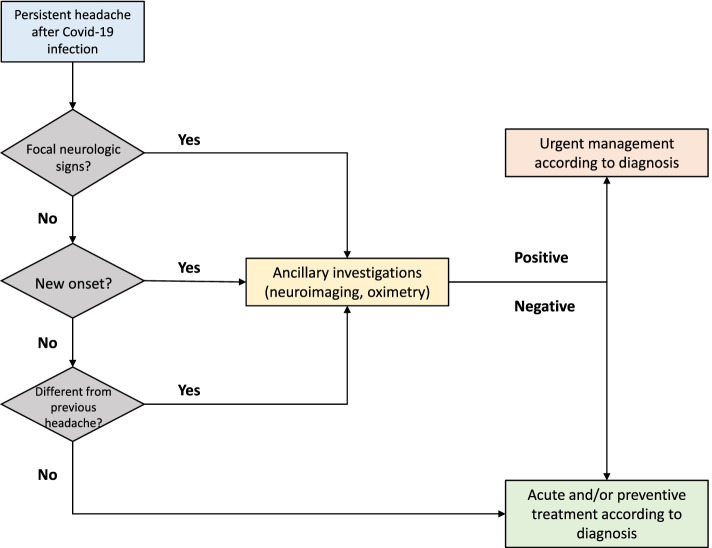
A proposed algorithm for the diagnosis of long COVID headache

The ‘red flags’ usually adopted for the diagnosis of secondary headaches should be considered to reveal possible cerebrovascular complications from COVID-19 or other secondary headaches, such as from ischemic or hemorrhagic stroke [52]. The presence of focal neurological signs together with headache should be accurately investigated as it may signal serious clinical conditions (potentially or not) related to COVID-19 infection [53, 54]. Some of those conditions may present with headache only; therefore, it is important to consider neuroimaging studies in elderly subjects with new-onset headache after COVID-19 infection. Furthermore, COVID-19 headache has other red flag signs including loss of smell and taste [53]. As COVID-19 infection could lead to persistent hypoxia, headache consequent to the infection itself should be distinguished from headache due to hypoxia or hypercapnia, even though both conditions may overlap [36]. Therefore, monitoring oxygen saturation should be considered, together with neuroimaging, when diagnosing COVID-related headaches. Figure 2 shows a proposed algorithm for the diagnosis of long COVID headache.

## Treatment approaches

Patients with long COVID headache require a multidisciplinary treatment approach, including pharmacological (acute and preventative) and non-pharmacological strategies [15]. There are, up to now, virtually no studies on long COVID headache treatment, so treatment recommendations are mainly based on existing recommendations for primary headache disorders, including migraine, tension-type headache (TTH) and NDPH. Treatment should take into account headache phenotype (migrainous vs. tension type like), comorbidities, and if present, additional post COVID-19 symptoms such as insomnia, mood disorders and cognitive difficulties [55].

### Pharmacological treatment

#### Tension-type headache (TTH)-like phenotype

Current guidelines on TTH treatment recommend simple analgesics (e.g. paracetamol) and non-steroidal anti-inflammatory drugs (NSAIDs) as first choice for acute treatment, followed by combination preparations that include caffeine [56]. It is important to educate patients about the frequency of use, to avoid the development of medication overuse headache. For prophylactic treatment, the tricyclic antidepressant amitriptyline is considered the drug of choice, followed by venlafaxine or mirtazapine, if no therapeutic effect is observed [56]. Treatment with amitriptyline can also help improve sleep quality, which can be particularly useful as long COVID headache is often accompanied by sleep disruptions [57].

Additionally, some data report benefits of glucocorticoids for the treatment of long COVID headache, in term of reduction of headache frequency and symptom intensity [20, 58].

#### Migraine headache-like phenotype

Similar as with the TTH-like phenotype, NSAIDs can be prescribed for the acute treatment of migraine-like attacks [59]. However, it is worth mentioning that a recent study reported three patients with migrainous long COVID headache, who were non-responsive to simple analgesics and/or NSAIDs [46]. A retrospective study analyzed the efficacy of indomethacin (50 mg, BID) in patients with long COVID headache, refractory to treatment with NSAIDs and triptans, and showed that 95% of patients reported greater than 50% headache relief from the third day of treatment, however, the long-term course is not reported [60].

Triptans have been considered as acute therapeutic options [55]. Caronna et al. reported two female patients (one with history of migraine), that were effectively treated with triptans [46]. Interestingly, in the same report, a male patient diagnosed with new onset hypertension and no previous history of migraine, was not responsive to any acute (or preventative) treatment. This could point to a difference in therapeutic efficacy profiles, based on the medical history of the patients and the concomitant post COVID-19 symptoms. However, a definitive therapeutic conclusion cannot be made on the basis of the limited data available.

For the prophylactic treatment, antidepressants and onabotulinumtoxinA (BTX) have been suggested [46, 55]. Caronna et al. have found that amitriptyline 25 mg QD in combination with BTX 195U resulted in an improvement of sleep quality, and a reduction in headache frequency [46]. Moreover, a small study showed that after six months of BTX treatment following the PREEMPT protocol (155–195U), a reduction in headache frequency and severity was achieved in patients diagnosed with NDPH like symptoms, a disorder characterized by persistent headaches [61], as seen in long COVID headache [62]. In addition, two retrospective studies with pediatric NDPH [63] and hospitalized COVID-19 patients non-responsive to paracetamol [32] suggest that greater occipital nerve block could also be used as short-term preventative treatment.

### Considerations

As mentioned above, to choose the best therapeutic option, it is necessary to evaluate the presence of other post COVID-19 syndrome symptoms. It is important not to discard the development of new onset or the worsening of an existing, cardiovascular disorder [57], as well as the presence of risk factors (e.g. obesity). In these cases, antimigraine drugs with vascular effects (e.g. triptans) should be avoided, due to their potential vasoconstrictive effect [64]. The protective role of CGRP in the maintenance of cardiovascular homeostasis, and in tissue remodelling in pulmonary hypertension [65, 66], should be instead taken into consideration.

Longitudinal studies that address efficacy and safety of current antimigraine treatments in long COVID headache are urgently needed. Moreover, potential therapeutic profiles, based on the previous medical history, post COVID-19 symptoms, and refractoriness to treatment would greatly optimize the pharmacological treatment of long COVID headache.

### Non-pharmacological treatment

Non-pharmacological treatment recommendations for post COVID-19 are mainly based on expert opinion. They include patient education with recommendations for lifestyle changes, physical therapy, psychological therapy and the management of pre-existing comorbidities [15]. Patient education should be directed towards a healthy balanced diet, a regular sleep–wake rhythm, and regular physical exercise [67]. Recommended physiotherapeutic exercises comprise both aerobic and strength training, but also training in breathing and relaxation techniques [68]. The intensity of physical exercise should be moderate and gradually increased because too intense exercise might exacerbate symptoms in individuals with post COVID-19 myalgic encephalomyelitis/chronic fatigue syndrome [69]. In case of high psychosocial burden or psychiatric comorbidities, cognitive behavioral therapy should be considered [67]. All these measures might be included in rehabilitation programs that can be carried out either in a clinical setting or autonomously at home. The WHO provides useful advice for patients on self-management after COVID related illness to improve self-rehabilitation and recovery (https://www.who.int/publications/m/item/support-for-rehabilitation-self-management-after-covid-19-related-illness). None of these suggestions specifically address long COVID headache but rather the whole long COVID symptom constellation. However, many of these strategies have been proven helpful in the management of headache disorders [70] and are likely to be effective against long COVID headache.

Regular follow-up consultations and the establishment of an empathic, understanding therapeutic relationship might also contribute to a general symptom improvement in all patients and especially in the long COVID population [71–73]. Group therapies and peer-support programs, both in person and virtual, provide further social, emotional, and informational assistance for the affected individuals [74, 75].

## Future directions and outlooks

The presence of headache should be screened in all patients with long COVID headache, and whenever present, healthcare providers, from primary care to specialized care, should ensure an adequate evaluation of patients. Current treatment strategies are based on existing recommendations for other headache disorders. Treatment studies, including randomized controlled multicentre trials, are urgently needed. At the moment, clinicaltrials.gov lists only one study, investigating the effect of sphenopalatine ganglion block with local anesthetics on long COVID headache (https://www.clinicaltrials.gov/ct2/show/NCT04636034). Treatment resistant patients or cases with atypical features, red flags or relevant comorbidities may benefit from specialized care, being most cases suitable for treatment in primary care setting.

Long COVID headache also has the potential to significantly advance headache research in several respects.

First, daily persistent long COVID headache bears significant similarities with one of the least understood headache types, NDPH [12, 76]. Although NDPH is classified among the primary headache disorders [11], part of the cases occur in association with viral infections (especially EBV) [77]. An immediate type (starting during acute infection) and a delayed type (starting weeks later) have been described [58, 77]. Immediate and delayed types of NDPH exist in long COVID headache [46]. In addition, both NDPH and COVID-19 associated headache can manifest with migrainous or TTH-like phenotypes [25, 77]. Historical reports suggest that persistent, daily headache was also a major health problem after the Russian/Asiatic flu (1889–1892) [18], further corroborating the link between viral infections and NDPH. Moreover, both NDPH and acute COVID-19 headache developing into long COVID headache are often treatment resistant [30]. Investigating daily persistent headache after COVID-19 may therefore be a good opportunity to better understand NDPH. It is an interesting question if immediate and delayed headache have different mechanisms. Understanding mechanisms may also allow development of treatments. The hypothesis of an ongoing meningeal immune reaction has prompted treatment with short courses of high-dose glucocorticoids in infection-associated NDPH [58], a strategy that has also been proposed for long COVID headache [20].

Second, the fact that long COVID headache can have a migrainous phenotype in patients without migraine history may stimulate research on shared mechanisms of primary and secondary headache disorders [30]. For example, the trigeminovascular activation leading to migrainous headache might be the result of genetic and environmental/psychosocial factors in primary headache disorders and of external factors such as infections or immune responses in secondary headache disorders. Indeed, migrainous phenotypes have also been described in other secondary headaches such as posttraumatic headache [78]. In addition, investigating long COVID headache can also promote our understanding of the role of chronic inflammation in headache.

Third, headache often comes as part of the long COVID syndrome, with additional symptoms like fatigue, insomnia, and depression [69]. This may be relevant, because in some cases, the selected treatment may also be beneficial for these (e.g., amitriptyline), while in other cases, patients may need specific treatment and/or evaluation for their comorbidities. Long COVID is a broad disorder where multi-disciplinary management is key for the adequate treatment of patients.

## Conclusion

There is a growing interest in long COVID headache. Studies have highlighted the need for specific recommendations and a tailored approach, although to date, most recommendations are given on the basis of the evidence available for primary headaches with same clinical characteristics. The diagnosis is based on the temporal relationship with infection from SARS-CoV-2 and exclusion of other secondary headaches. The presence of headache must be assessed in every long COVID case, and in the absence of atypical features or red flags, treatment can be started in primary care setting. Treatment resistant cases should be referred to specialized care, to avert the clinical burden of the disease and its impact in terms of disability, loss of productivity and psychological comorbidities.

## Data Availability

Not applicable.
